# 

**Published:** 1884-07

**Authors:** 


CONTENTS.

| | Page. | | Page. |
|---|---|---|---|
| Watching the Operations of Nature. .. | 119 | Forty Years Ago | 129 |
| Koumiss, or Araby Milk | 120 | A Famous Poem | 130 |
| Taking Medicines | 122 | Byron's Last Moments | 131 |
| Loss of Form | 123 | Modern Sources of Pin Money . ..... | 131 |
| Tar Smoke for Diphtheria | 124 | Russian Petroleum | 133 |
| Medicinal Virtues of Warm Milk | 124 | Remedy for Rheumatism | 134 |
| Dog Bites | 125 | Poisonous Plants and Flowers | 134 |
| A Nation of Gluttons | 126 | Origin of Some Nursery Rhymes | 135 |
| Grafted Teeth | 126 | What a Volcano Can Do | 137 |
| High Heels | 127 | Grapes | 137 |
| Female Tenacity of Life | 128 | Water | 138 |

